# Endoscopic resection of forehead lipoma: A subperiosteal single-portal approach

**DOI:** 10.4103/0970-0358.39665

**Published:** 2008

**Authors:** Terence Goh L. H., Bien-Keem Tan, Foo Chee Liam

**Affiliations:** Department of Plastic Surgery, Singapore General Hospital, Outram Road, Singapore, 169 608

**Keywords:** Endoscope, forehead, lipoma, subperiosteal

## Abstract

Forehead lumps which are commonly excised for cosmetic reasons, are done through a traditional open excision technique. In the Asian population, open excision commonly results in a pigmented scar which tends not to fade with time and this can be a concern to some patients. The aims of this paper are to describe an endoscopically assisted technique of surgery, with emphasis on the subperiosteal, single-portal approach; to review the outcome of the surgery, and to define the indications for its use.

**Materials and Methods:**

We describe here a case series of ten patients, in whom a minimally invasive technique was employed for the removal of a forehead lipoma *via* an endoscopic, single-portal, subperiosteal approach.

**Results:**

The patients' age ranged from 26 to 54 years and the dimensions of the masses removed ranged from 1.0 × 0.5 cm to 3.0 × 3.0 cm. All were confirmed by histological examination to be lipomata. The patients were followed up for an average period of nine months. There were no residual masses or recurrences and no complications of nerve damage. All the patients were very satisfied with their ‘scar less’ operations.

**Conclusions:**

Endoscopic excision of forehead lipomas through a single-port approach is both safe and reliable. It is indicated in patients who are prone to scarring or who are concerned with a forehead scar.

## INTRODUCTION

Forehead lipomas are common and are regularly removed *via* an open excision by general surgeons. Occasionally, a subset of these patients is referred to the plastic surgeon because the patient is concerned about scarring in the forehead.

Since 2006, we embarked on a minimally invasive technique for the removal of forehead lipomas through an endoscopic approach for this group of patients.

The aim of this paper is to raise the awareness of the use of the endoscopic approach for the removal of forehead lipomas in this region and to highlight the refinements made to existing techniques, namely: (1) single scalp incision approach, (2) use of the artery forceps as a dissector and grasper, and (3) subperiosteal dissection approach for forehead lipomas.

## MATERIALS AND METHODS

A total of ten patients were operated on between January and December 2006. Preoperatively, the patients were diagnosed to have a lipoma on clinical examination with the presence of a ‘slip’ sign.

A standard video system consisting of an endoscopic camera, xenon light source, television monitor, and video recorder was used for each procedure. The instruments included a 4.0 mm, 30 degree scope, retractor, periosteal elevators, and dissecting artery forceps.

### Operative procedure

**Figure 1:**
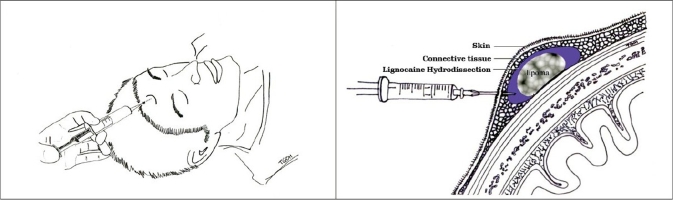
Hydro dissection with lignocaine to facilitate easy excision of the lipoma

The operation is performed under general anaesthesia. The procedure can also be done under local anaesthesia with conscious sedation. Prior to intubation, the forehead lump is marked with a surgical marker and the patient is anaesthetised. The next step, which is also the most crucial, is to perform hydro dissection by injecting lignocaine and adrenaline to ‘free’ the lump from the surrounding tissue [Figure 1] The injection is given circumferentially as well as above and below the lump and along the tract for dissection. This helps to create a bloodless field for the introduction of the endoscopic instruments, and frees the lipoma from the surrounding skin and subcutaneous attachments, making dissection easier.

**Figure 3:**
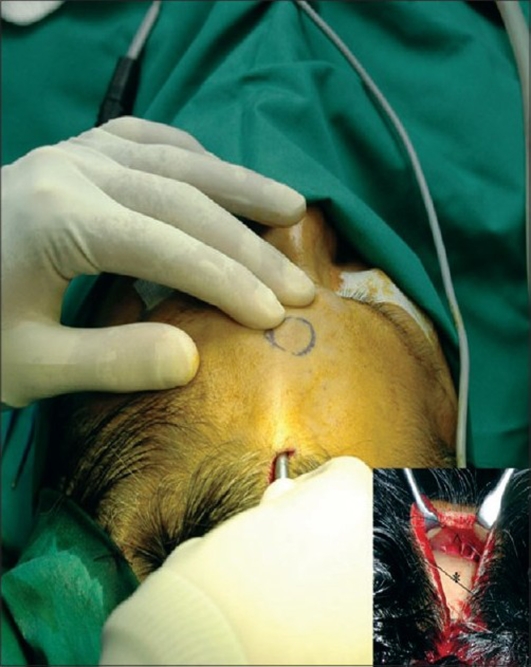
Subperiosteal plane revealing periosteal layer (^∧^) (above) and calvarium (*) (below)

A sterile comb is used to part the hair to avoid shaving the incision site. In males, future balding is taken into consideration and the incision is placed a bit farther back from the current hairline. A single 2.5 cm parasagittal incision is located approximately 2–3 cm behind the hairline. The incision is bevelled and placed parallel to the hair follicles so as to reduce the chance of alopecia. The skin incision is made with an 11-blade past the hair follicles and deepened with the Colorado needle down to the calvarium. Next, a periosteal elevator is used to dissect sub periosteally until the base of the lump is visualised [Figure 2]. This plane is relatively bloodless [Figure 3] and the endoscope and the dissecting forceps are introduced through the same incision.

**Figure:**
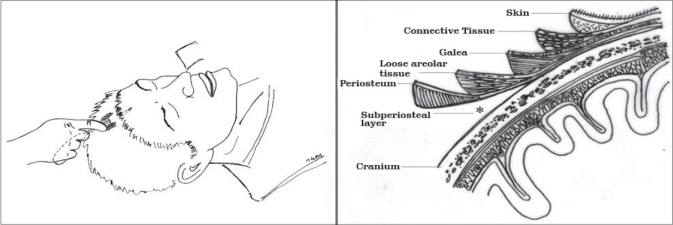


**Figure 4:**
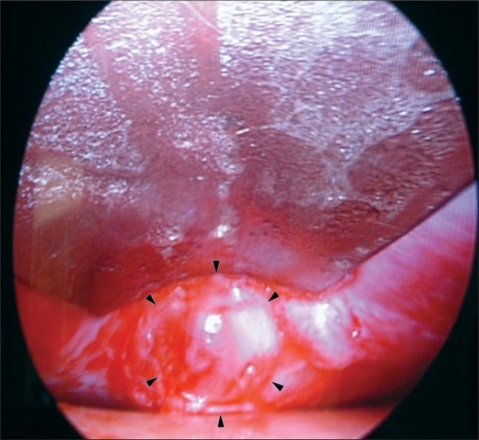
Endoscopic view of the lipoma (arrows)

The precise location of the lipoma on the inside can be confirmed by pressing on the lump externally. A rent is then made in the periosteum and galea using a pair of artery forceps, which will bring the lipoma into view [Figure 4].

Due to prior local anaesthetic infiltration, minimal dissection is required to free the lump from the surrounding tissues. The lipoma is easily removed by “pushing and pulling”, which is, finger pressure on the outside coupled with dissection and traction from the inside.

The wound is closed primarily with Vicryl to the subcutaneous layers and staples to the scalp. A drain is inserted which will be removed the next day. A pressure bandage is applied around the forehead.

## RESULTS

**Figure 5:**
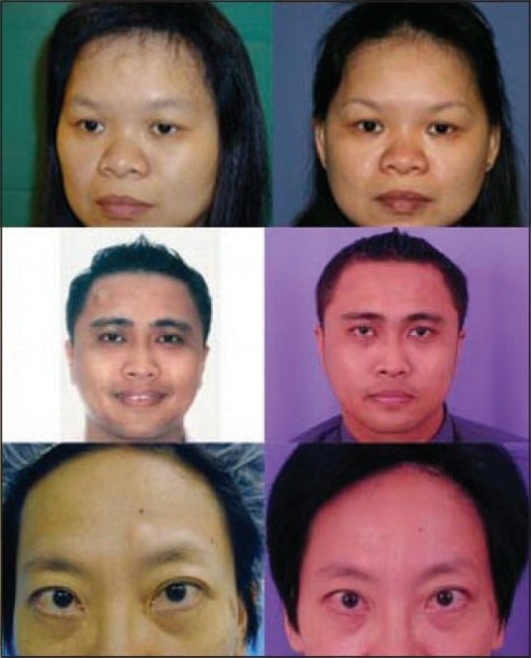
Pre- and postoperative photographs

There were seven female and three males in our series [Table 1]. Their ages ranged from 26 to 54 years and the size of the lipomata ranged from 1.0 × 0.5 cm to 3.0 × 3.0 cm. All lumps were confirmed by histological examination to be lipomas. The duration of surgery ranged from 20 to 60 minutes with an average of 45 minutes per procedure. There was no need for conversion to open surgery. All patients were discharged on the day of the surgery and reviewed in the clinic on the following day. There were no complications of residual mass, haematoma formation, postoperative swelling, or scalp anaesthesia. All patients were very satisfied with the procedure [Figure 5].

**Table 1:** Results of endoscopic lipoma excision via single-port technique

*Age*	*Sex*	*Race*	*Duration*	*Size (cm)*	*Location*
54	F	Chinese	40	1.0 × 0.9	Forehead
39	F	Chinese	45	1.0 × 0.6	Forehead
40	F	Chinese	60	1.5 × 1.0	Forehead
38	F	Filipino	50	1.2 × 1.2	Right Brow
38	M	Chinese	45	1.0 × 0.6	Forehead
26	M	Malay	20	1.0 × 1.0	Forehead
51	F	Chinese	45	1.3 × 0.7	Right Brow
44	F	Chinese	60	3.0 × 3.0	Forehead
41	M	Chinese	40	1.0 × 0.8	Forehead
42	F	Chinese	40	1.6 × 1.1	Forehead

## DISCUSSION

An endoscopic approach allows excision of the forehead lump through scars hidden in the scalp. More importantly, it is safe and allows the surgeon to operate under magnification, thus reducing the risk of injury to the neurovascular structures of the forehead.

Our present technique employs a single-port approach which reduces the number of scars over the scalp as well as the risk of alopecia. Other surgeons[1,2] prefer two or three ports to avoid entanglement of the instruments. This is well circumvented by using a single artery forceps which fulfils the role of a dissector as well as a grasper.

The subperiosteal plane is preferred as it is relatively bloodless and easy to access in the region of the forehead and there were no problems with exposure. With digital pressure, the lump can be located easily and a rent is made in the periosteal and galeal layers. The supraorbital and supratrochlear nerves traverse in the subgaleal layer and are hence, protected from the subperiosteal dissection,[3,4] avoiding the need to make any special effort to identify the nerves. It has been found that subperiosteal dissection is more reliable in preserving sensation in the forehead.[3,4] Additionally, subperiosteal dissection also preserves the subgaleal blood supply to the scalp.[3] In our experience, bleeding is minimal and hemostasis is rarely required.

General endoscopic equipment used by orthopaedic surgeons, otolaryngologists, and general surgeons can be adapted for use in this procedure and would not incur any significant cost to the patient. The only additional costs are for the increased operating time and the use of general anaesthesia. The operating time is longer because of the indirect approach and we have found that although the operation can be performed under local anaesthesia, patients are more comfortable with the procedure done under general anaesthesia.

All our patients are very satisfied with the outcome of the surgery. Our experience supports the use of endoscopic approach for removal of forehead lumps.
